# Identification of adequate vehicles to carry nerve regeneration inducers using tubulisation

**DOI:** 10.1186/1471-2202-13-100

**Published:** 2012-08-14

**Authors:** Adriana Helena do Nascimento-Elias, Bruno César Fresnesdas, Maria Cristina Lopes Schiavoni, Natália Fernanda Gaspar de Almeida, Ana Paula Santos, Jean de Oliveira Ramos, Wilson Marques Junior, Amilton Antunes Barreira

**Affiliations:** 1 Department of Neurosciences, School of Medicine of Ribeirão Preto, University of São Paulo (USP), Ribeirão Preto, São Paulo, Brazil; 2 Federal University of Jequitinhonha and Mucuri Valleys (UFVJM), Diamantina, Minas Gerais, Brazil; 3 Departamento de Neurociências, Faculdade de Medicina de Ribeirão Preto, Av Bandeirantes 3900, CEP: 14049-900, Ribeirão Preto, São Paulo, Brasil

**Keywords:** Pharmaceutical vehicle, Agarose, Culture medium, Collagen, Nerves, Peripheral nerves, Nerve regeneration, Neuronal plasticity, Sciatic nerve, Wallerian degeneration

## Abstract

**Background:**

Axonal regeneration depends on many factors, such as the type of injury and repair, age, distance from the cell body and distance of the denervated muscle, loss of surrounding tissue and the type of injured nerve. Experimental models use tubulisation with a silicone tube to research regenerative factors and substances to induce regeneration. Agarose, collagen and DMEM (Dulbecco’s modified Eagle’s medium) can be used as vehicles. In this study, we compared the ability of these vehicles to induce rat sciatic nerve regeneration with the intent of finding the least active or inert substance. The experiment used 47 female Wistar rats, which were divided into four experimental groups (agarose 4%, agarose 0.4%, collagen, DMEM) and one normal control group. The right sciatic nerve was exposed, and an incision was made that created a 10 mm gap between the distal and proximal stumps. A silicone tube was grafted onto each stump, and the tubes were filled with the respective media. After 70 days, the sciatic nerve was removed. We evaluated the formation of a regeneration cable, nerve fibre growth, and the functional viability of the regenerated fibres.

**Results:**

Comparison among the three vehicles showed that 0.4% agarose gels had almost no effect on provoking the regeneration of peripheral nerves and that 4% agarose gels completely prevented fibre growth. The others substances were associated with profuse nerve fibre growth.

**Conclusions:**

In the appropriate concentration, agarose gel may be an important vehicle for testing factors that induce regeneration without interfering with nerve growth.

## Background

More than 600,000 people in the United States and the European Union are afflicted with accident-related nerve injuries each year, and this presents a serious public health concern and a loss of quality of life for affected people [1]. The transection of nerves is followed by Wallerian degeneration [2], which is a complex biologic process beginning with the release of proteases that digest the myelin sheath and the axon in the distal stump of the nerve [3]. Axonal regeneration depends on many factors, such as the type of injury and repair, age, distance from the cell body and distance of the denervated muscle, loss of surrounding tissue and the type of injured nerve. Complete regeneration of a peripheral nerve can be slow and may even take years, and the recovery of motor function may be incomplete or absent [4]. Loss of tissue caused by trauma- or disease-induced nerve disruption is a significant limiting factor in nerve regeneration. To overcome this kind of difficulty, it became necessary to develop specific surgical techniques to facilitate nerve regeneration and eliminate the damage caused by the absence of tissue [5]. The use of hollow structures as a bridge between nerve stumps is one of those techniques. In this technique, tubes of synthetic or natural materials are used to guide the growth of axons. Even though the tubes are empty, they can facilitate recovery when the gap is of sufficient length because neurotrophic factors produced by the proximal stump can accumulate in the interior of the tubes [6]. Tubulisation with a silicone chamber is used in experimental models for research on regenerative factors because the silicone provides an isolated and inert environment that impairs the entry and exit of large molecules and is not itself absorbed. For some, these features make silicone chambers ideal for the study of the effect of different substances on nerve regeneration [7]. The tubes can also be filled with different substances that can stimulate regeneration such as neurotrophic factors [8], proteins that promote the synthesis of DNA, stem cells [9], and Schwann cells [10]. However, regeneration-inducing substances require the use of vehicles. Agarose is one substance that can be used as vehicle. It is a natural polysaccharide that is derived from red algae, and it is composed of subunits of galactose. When dissolved in hot water, agarose acquires a gelatinous consistency and forms a hydrogel [11,12]. Collagen can also be used as a vehicle or as a luminal additive, and it is widely used in studies of nerve regeneration – it is even used in the production of absorbable tubes – with varying degrees of success [13,14]. DMEM (Dulbecco’s modified Eagle’s medium) has been used as a culture medium for stem cells and has therefore also been used as a vehicle in nerve regeneration studies using stem cells. To check the usefulness of DMEM in the induction of regeneration, it is important [15] to keep in mind the potential ways that it can induce regeneration.

An inert vehicle that does not inhibit or induce nerve regeneration would allow for a better evaluation of inducers of nerve regeneration. In this study we attempted to identify an inert or minimally active vehicle by comparing the ability of agarose (0.4% and 4%), collagen type I (3 mg/ml) and DMEM to induce rat sciatic nerve regeneration.

## Results

**Figure 1:**
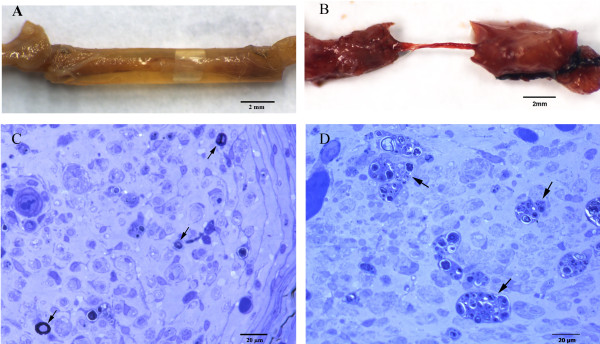
**Tubulisation and microscopic aspects of the distal segments in nerves of 0.4% agarose treated rats 70 days after.** (**A**) Regeneration cable within the tube of a 0.4% agarose-treated rat. Agarose gel in this concentration is not a barrier to the cable growth. Left = proximal. Right = distal. Bar = 2 mm, (**B**) Filiform cable in a rat nerve treated with DMEM. Left = proximal. Right = distal. Bar = 2 mm, (**C**) Three myelinic fibres in the distal segment of a 0.4% agarose-treated rat (arrows). Semithin section (0.5 μm). Toluidine blue. Bar = 20 μm, (**D**) Endoneurial area of the distal sciatic nerve segment from an animal treated with 0.4% agarose. The presence of some digestion chambers filled with myelin debris indicates that Wallerian degeneration occurred (arrows). Semithin section (0.5 μm). Toluidine blue. Bar = 20 μm.

There was no appreciable regeneration in nerves treated with agarose. None of the animals treated with 4% agarose formed cable regeneration in their nerves. In nine of the fifteen animals treated with 0.4% agarose, a connection between the stumps was formed. In seven animals, the macroscopic aspect of the regeneration cable was similar to normal sciatic nerves (Figur 1A), and a filiform cable was formed in the nerves of the other two rats. In the group of animals treated with DMEM, there was no connection between the nerves in two of the eight rats, and there was a thin regeneration cable between the two stumps in the remaining six rats (Figure 1B). In the group treated with collagen, a connection cable grew in seven animals. Three cables were thin, and four cables were macroscopically similar to those observed in normal untreated animals. For all groups, the only difference in the cable between treated and normal nerves was a thinner central portion in comparison with the diameter of the portion closer to stumps.

The average fascicular area, the number and density of fibres of groups III, IV and the control group and the fascicular area of group II are all shown in Table 1. Six of the animals from the group that received 0.4% agarose exhibited nerve fibre regeneration. In three of those animals, a few nerve fibres (no more than 30) reached the distal segment (Figur 1 C). In the other three animals, nerve fibres (no more than 150) reached the middle of the cable. The presence of some digestion chambers with myelin debris indicated that Wallerian degeneration had occurred in distal segment fibres (Figur 1D).

**Table 1:** Morphometric aspects

**Group**	**Fascicular area (mm²)**	**Fibre numbers**	**Density (n°/mm²)**
Collagen	0.52 ± 0.4	2241.0 ± 4197.5	3193.8 ± 8538.3
DMEM	0.46 ± 0.3	3356.6 ± 2868.3	7434.4 ± 5409.6
Control	0.64 ± 0.1	7878.0 ± 474.6^**^	11935.1 ± 518.9
Agarose 0,4%	1.40 ± 0.3^*^	-	-

* Indicates a significant difference between the 0.4% agarose group and the other groups (p < 0.05).

** Indicates a significant difference between the control group and the other groups (p 1< 0.05).

The histogram of normal sciatic nerves showed a peak fibre diameter of between 4 μm and 6 μm (Figur 2A). The fibres of the sciatic nerve from normal animals reached a maximum of 10.3 μm in diameter, the nerve fibres from the animals treated with collagen reached a maximum of 8 μm in diameter (Figur 2B) and the fibres of the animals from the DMEM group reached a maximum diameter of 8.5 μm (Figur 2C). Histograms of the axonal diameters of the sciatic nerve from normal rats and from rats from groups III and IV are also shown in Figure ( 2D, 2E and 2F).

**Figure 2:**
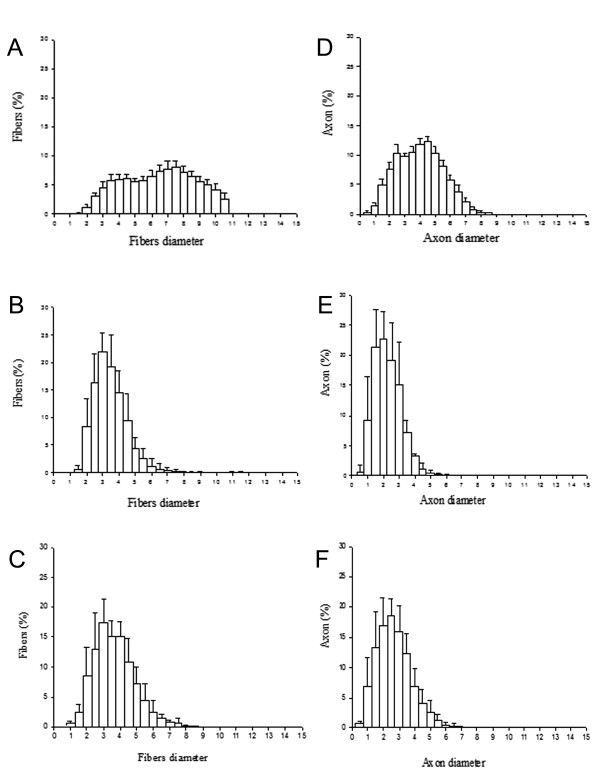
**Histograms of myelinated fibres and axons of sciatic nerves.** (**A**) Control side, (**B**) rats treated with collagen, (**C**) DMEM group, (**D**) axons from control animals, (**E**) collagen-treated group, (**F**) DMEM-treated group.

**Figure 3:**
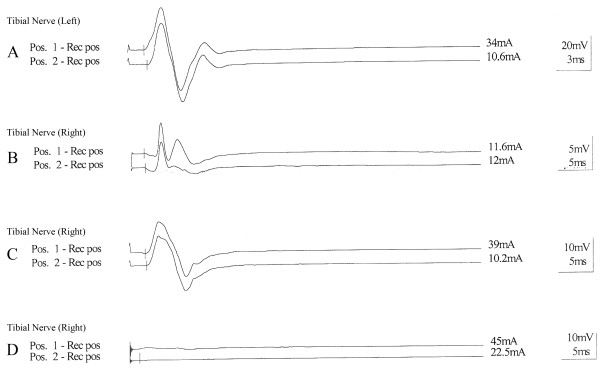
**Electrophysiological recordings from rat sciatic nerve branches.** (**A**) Normal, (**B**) collagen, (**C**) DMEM, (**D**) 0.4% agarose. The record electrode was placed in the gastrocnemius muscle motor point. (A) Normal, (B) collagen, (C) DMEM, (D) 0.4% agarose.

There were no significant differences in the amplitude of the compound muscle action potential (CMAP) and in the conduction velocity between the collagen and DMEM groups. However, both of those parameters were statistically different between the collagen and DMEM-treated groups and the control groups. The average conduction velocities of the collagen, DMEM and control groups were 40,0 (± 7.7), 33.3 (± 10.4) and 54.4 (± 13.0) m/s, respectively, and the average CMAP values for the collagen, DMEM and control groups were 3.0 (± 2.0), 3.3 (±7.0) and 32.5 (± 5.9) mv, respectively. No nerve potential was obtained in the electrophysiological studies of the 4.0% and 0.4% agarose groups (Figure 3).

## Discussion

In recent years a multimodal approach to study nerve regeneration has progressively being used. Microsurgery, cell and tissue transplantation, material science, and gene transfer can be combined under the designation of tissue regeneration [16]. Although different possibilities in each approach of tissue regeneration are being explored in many research laboratories, the pursuit or adequate vehicles to transport inducers of nerve regeneration has been neglected. In the present study, it was determined whether 4% and 0.4% agarose, DMEM and collagen could act as vehicles for inducers of regeneration of the sciatic nerve of Wistar rats by placing them in a silicone tube that was positioned in the 10 mm gap that joined the stumps. In 40% of the animals, 0.4% agarose was not capable of inducing the growth of an adequate extracellular matrix, and even when an extracellular matrix was formed, only a few of the nerve fibres displayed regeneration. DMEM and collagen induced the growth of an extracellular matrix and promoted the regeneration of electrophysiologically viable nerve fibres, which indicated that there is a high probability that some clinical functional viability of the paw would occur after longer periods of treatment. Interest in the search for vehicles that support inducers of regeneration has increased in recent years because an extensive variety of substances, growth factors and cells obtained from cultures that are active for nerve regeneration have been tested [17-23], and it is important to know if the vehicle itself is active or if its interaction with the inducer of regeneration is active.

A previous study showed that 30 days after tubulisation, 60% of rats showed almost no regeneration of the sciatic nerve fibres in the 10 mm gap between the proximal and distal stumps, although a regeneration cable grew in all of the animals. Nerve fibre regeneration occurred in 40% of the animals, but few axons reached the distal stump. When the gap was 15 mm or more, a regeneration cable was formed, but no fibres reached the distal stump [23]. In a study using tubulisation with a gap of 20 mm where agarose was used as a vehicle for different concentrations of nerve growth factor and laminin-1 for the experimental group and 0.5% agarose was used alone in the control group, no nerve fibres grew in the control group [11]. The results of the present study in which 0.4% agarose was used are similar to the results of the aforementioned study [23], which used an empty tube and a tube filled with 0.5 agarose and a smaller observation time. This indicates that the effects of a 0.4% agarose-filled tube that joins the sciatic nerve stumps with a gap of 10 mm can closely mimic the effects of an empty tube in a similarly sized gap. Studies that used a 4 mm gap and that were based on clinical and electrophysiological evaluations and quantifications of pharmacologically induced sweat paw pads in mice indicated that 2%, 1% and 0.5% concentrations of agarose are increasingly capable of inducing nerve regeneration [24]. These findings were confirmed in another study by *in vitro* observations, and this indicates that 1% and 0.5% agarose concentrations cause a growth of neuritis. No neuritis growth was observed when using a 1.25% or higher agarose concentration [25]. These *in vitro* findings are corroborated by the results found here where 4% agarose was used. The prevention of neuritis and nerve fibre outgrowth that is associated with the use of a higher agarose concentration was attributed to the exponential reduction of the gel pore radius that was measured with electron microscopy. This reduction provides a physical barrier that impedes the growth of neuritis. *In vitro* concentrations of agarose of 4% or higher are associated with a lack of nerve fibre growth [26]. In the present study, a 10 mm gap was formed by a silicone tube placed between sciatic nerve stumps and filled with agarose gel. After 70 days, no regeneration cable was observed in the rats that had had their tubes filled with 4% agarose. In the 60% of the rats with tubes filled with 0.4% agarose, a regeneration cable reached the distal stump, and in 20% of them, no more than 30 fibres reached the distal stump. If this concentration of agarose is appropriately mixed with substances or active factors that are inducers of nerve fibre growth, agarose can be used as a vehicle because its viscosity allows the maintenance of continuous contact between those substances and the nerve stumps. This indicates that an agarose concentration of 0.4% or less can be a useful inactive or a poorly active vehicle in studies that use tubulisation to investigate inducers of nerve fibre growth in peripheral nerve regeneration. The results of the present study, which used agarose concentrations of 0.4% or less and which still preserved some degree of viscosity, indicate that this concentration of agarose can be a useful vehicle for testing specific inducers of regeneration because these inducers do not seem to interact chemically with agarose. The preservation of the gel consistency is obtained in concentrations of 0.12% or more [27]. In lower concentrations, agarose loses its gel consistency, and the use of concentrations higher than 0.12% is recommended because this concentration is considered the percolation threshold [28]. Considering the safety margin suggested by these authors and the fact that we used a concentration of 0.4% in this study, it is plausible to assume that concentrations between 0.2% and 0.4% agarose preserve the gel condition. In those concentrations, agarose is an inert or almost inert vehicle and does not induce the growth of any significant number of nerve fibres. It can therefore be used to test factors or substances that induce the growth of peripheral nerve fibres. In the present study, the absence of CMAP in nerves treated with 4.0% and 0.4% agarose supports the observation that there was no nerve fibre growth in the first group and indicates that the number of nerve fibres in the second group was so low that their potentials were not detectable. Vehicle fluids, such as normal saline, can eventually be lost over a time period of days, which means that the constancy of their contact with the stumps of the sectioned nerve and its growth cone is not guaranteed. In addition, PBS seems to have some of its own activity and may facilitate the induction of the growth of nerve fibres. As evidenced in this study, 0.4% agarose causes only a negligible growth of fibres. In a study in rats, saline was able to induce the growth of approximately 218 fibres; however, a gap of 8 mm rather than 10 mm was used, and the time of observation was 84 days [29].

The profusion of regenerated fibres at the end of this experiment shows that DMEM is very active in the regeneration of functionally viable nerve fibres. In two previous studies in rats, DMEM was used as a control for comparison with stem cells. As in this study, a 10 mm gap was used, but the epineurium of the nerve itself was used for the tubulisation, and the observation time was 90 days [29]. In the present study, silicone tubes were used, and quantification of the fibres was made in the distal segment of the nerve. This does not allow for a good comparison between our results and those of the studies discussed above. The minimal difference in the values of the CMAP observed in this study compared to the other study [30] may have been due to differences in methods, the weight of the animals and the time of observation between the two studies. In another experiment that used a gap and had a shorter observation time, DMEM was active but significantly lower numbers of fibres reached the distal segment as compared to the numbers of Schwann cells and stem cells that reached the distal segment. The same occurred with the nerve conduction velocity [22]. Studies in mice [15,20] and rabbits [31] also indicate that DMEM has a favourable effect on the regeneration of peripheral nerves. The present study shows that agarose is less active or not active in the induction of nerve growth as compared to DMEM. This is an indication that agarose can be a more suitable vehicle for studies of nerve regeneration.

In the present study, collagen induced the growth of approximately 44% of the total number of nerve fibres from normal sciatic nerves (CG), and the thickness of the regeneration cables were close to the thickness observed in normal controls. The ability of neural progenitor cells and bone morrow stromal cells to induce nerve regeneration when combined with collagen (mainly type I) was evaluated in models where tubulisation was used to induce nerve regeneration of the sciatic nerve of rats and rabbits [19,31]. Collagen is an inductor of neuritis and of axonal growth, and this effect is observed when collagen is added to the culture medium of the control groups from those two studies [32-34]. The observation time is an important factor in evaluating the effectiveness of collagen in peripheral nerve regeneration. When collagen was used in a 14 mm gap on a tubulised peroneal nerve, there was significant regeneration of nerve fibres, and functional recovery was observed [35]. In studies in mice [13,36], the effects of different concentrations of collagen (1.28 mg/ml, 1.92 mg/ml and 2.56 mg/ml) in 4 mm and 6 mm gaps were observed. The best functional recovery occurred in the animals that had 4 mm gaps and had been treated with the lower concentration of collagen, but even animals treated with the highest concentration of collagen showed some degree of functional recovery. The higher concentrations of collagen have a higher density and create a barrier that obstructs the passage of cells from the extracellular matrix and impedes axonal growth. When collagen concentrations of 0.4 mg/ml or higher were used *in vitro,* there was a larger reduction in the efficacy of the induction of fibre growth [34]. Despite the variability in the quality of results from different studies, which may have resulted from differences between methodologies, such as the size of the gap [23] and the permeability of the tube [37], and from differences in concentrations [13] and recovery time, it is clear that collagen is an inducer of nerve growth. The efficacy of regeneration provoked by collagen can be explained by the fact that it is a protein that is naturally present in various components of nerve fibres. Collagen is a component of the extracellular matrix. It is also present in the endoneurium, perineurium and epineurium of peripheral nerves, and it has an active role in the normal maintenance of the nervous system. Moreover, as one of the constituents of the extracellular matrix, it performs an important role in the synthesis of proteins such as fibrin and laminin [38].

## Conclusions

The comparison among the three vehicles showed that an agarose gel concentration of 0.4% is poorly efficient in the regeneration of peripheral nerves and that an agarose gel concentration of 4% completely prevents fibre growth because it is a physical barrier to that growth. Thus, a 0.4% agarose gel may be an important inert vehicle for testing the unique effectiveness of factors that induce regeneration. In the day to day practice of our laboratory we use as little as 0.1% agarose that maintains a viscosity degree near to gelification and is a poorer regeneration inducer compared with 0.4% agarose. The use of vehicles such as collagen and DMEM are also important, but their ability to induce regeneration should be considered when they are used in association with cells, molecules or other growth factors. The possibility that collagen, DMEM and agarose can synergistically affect the induction of peripheral nerve regeneration deserves further consideration. To test new regeneration inducers a gap of 15 mm or more (23) and using agarose in appropriate concentrations as vehicle in the control group seem to prevent any spontaneous or induced fibres growth reaching the distal stump after as much as 120 days of observation (24). Future studies may show the effects of the interaction of nerve fibre growth inducers in association with different vehicles, with consideration of the properties of every inducer, the vehicles, their interaction and the vehicle’s own action as a regeneration inducer, particularly in lower concentrations.

## Methods

### Animals

Forty-seven female Wistar rats (USP-FMRP, 250 g – 350 g) were studied and divided into five groups. The rats had free access to food and water. The study was approved by the institutional “Animal Experimentation Ethics Committee” at the Medical School of Ribeirão Preto.

### Surgical procedures and experimental groups

The animals were anesthetized with ketamine chlorydrate (75 mg/kg) and xilazina (15 mg/kg) via intraperitoneal injection. The right sciatic nerve was exposed and an incision was made, creating a 10 mm gap between the distal and proximal stumps. A silicone tube (14 mm in length x 1.47 mm inside diameter x 1.96 mm outside diameter; Degania Silicone®) was grafted onto the stumps using a 10–0 nylon suture, and the tubes were filled with the respective media (the media are described below in the description of the experimental groups). The distal end of the tube was inserted 0,5 mm proximally to the sciatic nerve trifurcation. The skin was closed with a 4.0 nylon suture, and chlorhexidine (Merthiolate®) was applied on the incision site. Clinical and electrophysiological evaluations were made before starting the experiment and immediately before nerve removal. The sciatic nerve was removed on the 70th day after tubulisation. *In situ* fixation was obtained using 2% glutaraldehyde in sodium cacodylate buffer (0.025 M, pH 7.4). After removal of the nerve, the animals were sacrificed using a higher dose of anaesthetics.

The animals were divided into 4 experimental groups and one control group. The silicone tubes for each group were filled with the following: Group I (GI), 30 μl of agarose hydrogel at a concentration of 4% (Agarose D1 Pronadisa®, n = 8); Group II (GII), 30 μl of agarose hydrogel at a concentration of 0.4% (Agarose D1 Pronadisa®, n = 15); Group III (GIII), collagen 3 mg/ml (purified bovine collagen solution, pureCol®, AdvancedBiomatrix®, n = 8); and Group IV (GIV), DMEM (n = 8). The Control group (CG) (“fifth” group) was composed of the 8 first rats belonging to the first group (agarose 4% group). These animals were subjected to a symmetric contralateral segmentation of the left sciatic nerve after 70 days of tubulisation of the right sciatic nerve (n = 8). The morphometric data obtained from those normal left sciatic nerve segments were taken to compare with the data from the other groups of the study.

### Electrophysiological studies

Nerve conduction studies were conducted using a portable Keypoint 4® equipament (Medtronic). Stimulation and recordings were made with subdermal needle electrodes. On the 70^th^ day, the animals were anaesthetised, and the sciatic nerve was exposed. The active recording electrode was placed in the gastrocnemius muscle motor point, while the reference electrode was placed on the 4^th^ finger at the distal tendon of the same muscle. Stimulation was carried on distally and proximally to the tube with the cathode always positioned distally to the anode. Stimulus duration was 0.1 ms and intensity was always supramaximal, although extra-care was taken to avoid stimulation of other structures. Gain was set between 0.5 to 10.0 mV/division, sweep velocity was 0,5 ms/division and band analysis ranged from 10 Hz to 10 KHz per sweep or 0.5 ms per division. Latency was measured from stimulus artefact to the initial negative deflection, while the amplitude was measured from the baseline to the negative peak. To calculate the conduction velocity (m/s), the distance between the two stimulated points was divided by the difference in their proximal-distal latencies.

### Histological evaluation and morphometry

Immediately after the electrophysiological studies, the connective cables bridging the two stumps were examined, and their diameters were measured and photographed.

The distal segment of the nerve was sectioned 1 mm after the distal limit of the tube, fixed in 2% glutaraldehyde, post fixed in 1% osmium tetroxide, subjected to progressive dehydration, embedded in Epoxy resin (Epon 812^®^), cut into 0.5 μm thick slices with a microtome (Carl Zeiss^®^, model G/214711) and stained with toluidine blue.

The digitalisation process was obtained using a Zeiss Axiophot light microscope with a motorised stage, a JVC TK1270 video camera, a computer and KS 400 (Kontron 2.0) software. The nerve fascicular area was delimitated, and images of the myelinic fibres were obtained. The image magnification was 640 × (1.6 × 100 × 0.5 × 8), and each image represented an area of 4,548.4 μm². All myelinic fibres where counted, and their density (fibres/mm²) was obtained. We used a semi-automatic morphometric analysis approach. The irregular and obliquely sectioned fibres were removed using the Erase command. Only circular fibres were measured automatically by the KS 400 software. At least 30% of the fibres of each nerve were measured, and the following parameters were obtained: minimal axonal diameter, minimal fibre diameter and myelin sheath area [39].

### Statistical analysis

All values are expressed as the mean ± standard deviation. The non parametric Kruskal-Wallis test was used to compare the groups, and Dunn’s post hoc test was used to verify the differences between groups. The differences were considered significant when p ≤ 0.05.

## Competing interests

The authors declare that they have no competing interests.

## Authors’ contributions

AHN-E participated in the design of the study, carried out the descriptive and morphometric evaluation of the agarose-treated Wistar rats and wrote the majority of the manuscript together with AAB. BCF performed the main part of the nerve morphometry and helped draft the manuscript. MCLS participated in the design of the study, performed part of the experiments and helped to carry out the descriptive and morphometric evaluation of nerves. NFGA performed experiments, participated in the morphometric and descriptive evaluation of the treated nerves and helped to draft part of the manuscript. APS performed experiments and participated in the morphometric and descriptive evaluation of the agarose-treated nerves. JOR participated in the electrophysiological evaluation and interpretation of the obtained data. WMJ designed the electrophysiological technique used in the study, participated in the electrophysiological evaluation and interpretation of the electrophysiological data and drafted the electrophysiological part of the manuscript. AAB conceived of the study, participated in the design and coordination of the study, supervised the collection of data and wrote the majority of the manuscript together with AHN-E. All authors read and approved the final manuscript.

## Acknowledgements

The authors thank Mr. Antonio Renato Meirelles e Silva and Ms. Aracy Edwirges Vieira da Silva Dias for technical assistance, and Mr. Geraldo Cássio dos Reis for statistical analysis. This work was supported by Fundação de Amparo à Pesquisa do Estado de São Paulo (FAPESP), Coordenadoria de Aperfeiçoamento do Pessoal do Ensino Superior (CAPES), Conselho Nacional de Pesquisa (CNPq) and Fundação de Apoio ao Ensino à Pesquisa e Assistência do Hospital das Clínicas da Faculdade de Medicina de Ribeirão Preto (FAEPA).

AHN-E is a PhD student. BCF is a Master’s student. MCLS is a Laboratory specialist. NFGA has a Master’s in Neuroscience. APS is an Associate Professor of Neurorehabilitation. JOR is a neurologist and electrophysiologist. WMJ is an Associate Professor of Neurology, Head of the Electromyography Unit of the University Hospital and a Peripheral Nerve Society member. AAB is a Full Professor of Neurology, Head of the “Laboratory of Experimental and Applied Neurology” and a Peripheral Nerve Society member.
